# Cause-specific mortality among patients with different molecular subtypes of T1-2N0M0 breast cancer: A population-based study

**DOI:** 10.1097/MD.0000000000027605

**Published:** 2021-10-29

**Authors:** Daoliang Wang, Liang Yi, Lijun Zhang, Zhuo Wang

**Affiliations:** a Department of Breast Surgery, The First People's Hospital of Jingzhou City, Shashi District, Jingzhou City, Hubei, China; b Department of Oncology Plastic Surgery, Hunan cancer Hospital, Hunan, China.

**Keywords:** breast cancer, cause-specific mortality, risk factors

## Abstract

The objective of our study is to investigate mortality pattern and quantitatively assess prognostic risk for cause-specific death among T1-2N0M0 breast cancer survivors.

The representative data of T1-2N0M0 breast cancer patients diagnosed between 2010 and 2016 was retrieved from the Surveillance, Epidemiology, and End Results program. Standardized mortality ratios (SMRs) were calculated taking US population as a reference. Cox regression analysis was conducted to analyze the potential prognostic factors for cause-specific mortality.

A total of 161,966 patients were identified from the Surveillance, Epidemiology, and End Results database. After a median follow-up of 41 months, mortality occurred in 10,567 patients, of which 30.9% and 22.7% were attributed to breast cancer and cardiovascular diseases (CVDs). The standardized mortality ratios of CVD were 4.78, 4.27, 3.78, and 4.95 in patients with HR+/HER2+, HR−/HER2+, HR+/HER2−, and HR−/HER2− breast cancer compared to general US population, respectively. Cox proportional hazards regression analysis showed that the adjusted HRs of breast cancer-specific mortality were 0.999 (95% confidence interval [CI]: 0.879–1.135), 1.454 (95% CI: 1.246–1.697), 2.145 (95% CI: 1.962–2.345) for HR+/HER2+, HR−/HER2+, and HR−/HER2− breast cancer, respectively, as compared with HR+/HER2− subtype; HRs of CVD-specific death were 1.215 (95% CI: 1.041–1.418), 1.391 (95% CI: 1.209–1.601), and 1.515 (95% CI: 1.213–1.892), respectively. In addition, we found that older age at diagnosis, and black race were also independent predictors of CVD-specific death.

In the present study, we revealed the mortality pattern of cause-specific mortality, and identified prognostic factors of overall mortality, breast cancer-specific mortality, and CVD-specific mortality in T1–2N0M0 breast cancer survivors, supporting early detection and more efficient CVD care for these patients.

## 1 Introduction

Breast cancer (BC) is the most frequent malignancy and the leading cause of cancer-associated mortality among women worldwide.^[1,2]^ The incidence rate of BC has slightly raised since 2004 (by approximately 0.3% annually), partially due to the increased obesity as well as continued declined in the fertility rate.^[3]^ In 2020, There will be an estimated incidence of 279,100 new cases and approximately 42,690 deaths in the United states.^[1]^ Fortunately, in the past few decades, due to the introduction of endocrine therapy, human epidermal growth factor receptor-2 (HER-2)-directed therapies and adjuvant chemotherapy, a significant improvement of survival had been witnessed in women with breast cancer, especially in those with T1–2N0M0 BC.^[4]^ Consequently, there are approximately 2,443,077 survivors who were diagnosed with female BC in the past 15 years in the United states.^[5]^ In addition, considering the steady uptrend of breast cancer incidence and the aging of population, the number of BC survivors will continue to increase, which necessitates a better understanding of health issues influencing survivorship.

Comprehensively understanding the patterns of cause of death (COD) and accurate prediction of cause-specific mortality are able to facilitate the improvement of timely intervention strategy and tailored surveillance for those vulnerable BC patients. Ursaru et al found that the most common cause of mortality in BC patients are metastasis to lung, liver, brain, and pleura.^[6]^ In addition, an observational study investigated the general causes of mortality in BC patients in European countries. The results showed that lymph node status is a critical predictor of favorable prognosis, and circulatory diseases are also an important cause of mortality, especially in BC women diagnosed at an older age.^[7]^ However, studies investigating the pattern of COD among patients with T1–2N0M0 BC remain limited.

In the present study, we retrospectively analyzed the mortality pattern of T1–2N0M0 BC patients and explored the prognostic factors of cause-specific death. This study may be valuable for clinicians to mitigate the risk of death and improve the overall survival rate among patients with T1–2N0M0 BC.

## 2 Methods

### 2.1 Data source and study population

In the present study, we conducted a retrospective analysis of data extracted from the Surveillance, Epidemiology, and End Results (SEER) database. Briefly, SEER, a registry affiliated to the National Cancer Institute, presently covers approximately 28% of the US cancer population.^[8]^ Using the SEER^∗^Stat (version 8.3.6), Patients aged 18 years or older with a histologically confirmed diagnosis of primary T1–2N0M0 breast cancer between 2010 to 2016 was included for further analysis. In addition, patients were excluded if diagnosed at death certificate or autopsy only, with >1 malignancy, without actively followed up, or missing information on any characteristics of interest including cause of death. Since all data of the SEER database were deidentified for public, our study was considered exempt from ethical approval.

The data collected from the SEER database included basic demographics (age, sex, race), pathological characteristics of tumor at diagnosis (AJCC TNM category, differentiation grade, disease subtype), and survival status. Since the human epidermal growth factor receptor 2 (HER2) status was only available in 2010 to 2016, for patients diagnosed between 2004 and 2009, the cancer subtype was classified as HR+ (progesterone receptor-positive and/or estrogen receptor-positive) and HR− (progesterone receptor-negative and/or estrogen receptor-negative). For those diagnosed between 2010 and 2016, the cancer type was classified as HR+/HER2+, HR−/HER2+, HR+/HER2−, or triple-negative (HR−/HER2−).

The primary outcome of our study was mortality of any cause. Follow-up period was cumulated from the date of initial diagnosis to death, or the last follow-up (December 2016). The *International Classification of Disease* codes were utilized to define cause of death for each patient, including BC-specific death, other cancer-specific death, cardiovascular death, and other noncancer death.

### 2.2 Statistical analysis

Descriptive statistics was performed to summarize data. The categorical data was presented with frequency and percentage. *χ*^2^ test was applied to assess statistical differences between groups. We then calculated standardized mortality ratio (SMR) with 95% confidence interval (CI) for each selected cause of mortality. SMR was defined as the ratio of the actual mortalities due to a specific cause in our study group to expected mortalities in general US population. The expected mortality represents the number of people who are expected to die from the same cause in a demographically alike population within the same timeframe.^[9]^ Cox proportional hazards regression model was applied to screen prognostic factors for all cause of death and cause-specific death.

All statistical analyses were performed with Microsoft Excel 15.0.4 (Micro- soft, Redmond, WA), SPSS version 22.0 (IBM Corporation, Armonk, NY), GraphPad Prism version 8 and R software (version 3.5.1). Tests were 2-side and *P* < .05 indicated a statistically significant difference.

## 3 Results

### 3.1 Characteristics of patient

In this study, 161,966 patients with T1–2N0M0 breast cancer were included for analysis. The demographic and clinicopathological features of these patients were summarized in Table 1. After a median follow-up of 41 months, mortality occurred in 10,567 patients, leading to an overall death rate of 6.52%. Patients who died had statistically significant proportion of cases with older age at diagnosis, higher grade tumor, and higher T stage than survivors.

**Table 1:** Demographic and clinicopathological characteristics of patients with T1–2N0M0 breast cancer.

Variables	All patients (%)		Overall death (%)	
N	161966		10567	
Age, y				
≤45	15545	9.6	455	4.3
45–65	78355	48.4	2347	22.2
65–85	61761	38.1	5529	52.3
>85	6305	3.9	2236	21.2
Race				
White	130709	80.7	8718	82.5
Black	15754	9.7	1298	12.3
Other	15503	9.6	551	5.2
Year at diagnosis				
2010–2012	76560	47.3	7581	71.7
2013–2016	85406	52.7	2986	28.3
Grade				
Well	46953	29.0	2409	22.8
Moderately	71565	44.2	4410	41.7
Poorly	43116	26.6	3705	35.1
Undifferentiated	332	0.2	43	0.4
T stage				
T1	120985	74.7	6286	59.5
T2	40981	25.3	4281	40.5
Molecular type				
HR+/HER2+	14873	9.2	838	7.9
HR−/HER2+	5577	3.4	383	3.6
HR+/HER2−	124540	76.9	7522	71.2
HR−/HER2−	16976	10.5	1824	17.3

### 3.2 Characteristics of cause-specific mortality

Of all mortalities in our study, 30.9%, 15.0%, and 54.1% were attributed to breast cancer, other tumors, noncancer causes, respectively. Lung and bronchus accounted for 25.6% of deaths attributable to other cancers, followed by miscellaneous malignant cancer (12.2%). Of noncancer mortalities, cardiovascular disease (CVD), including diseases of heart, hypertension, cerebrovascular disease, and atherosclerosis, was the most common cause, followed by diabetes (4.2%), accidents and adverse effects (3.8%).

**Figure 1:**
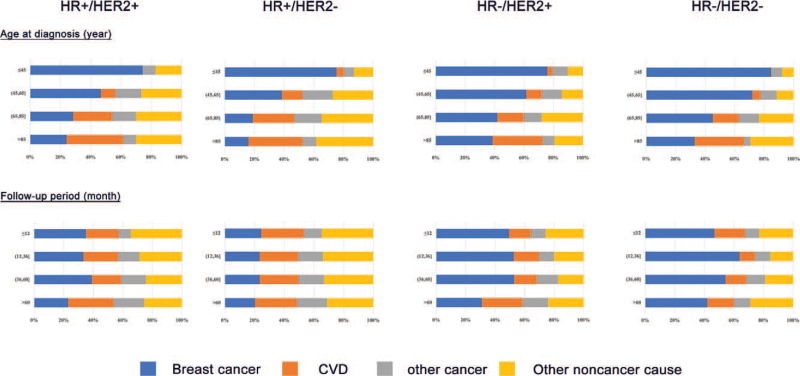
Proportional mortality ratio of cause-specific mortality, stratified by age at diagnosis and follow-up period in 4 molecular subtypes.

Figure 1 demonstrated the proportional mortality ratio stratified by patient demographics in various molecular subtypes. Breast cancer was the predominant cause of death within 1 year of diagnosis for all cancer subtypes and its proportion decreased over time. On the contrary, the proportional mortality ratio of CVDs gradually increased with time after diagnosis. A higher proportion of CVD deaths was also observed in older patients.

### 3.3 Risk of cause-specific mortality in patients versus general population

The relative risk of all-cause and CVD-specific deaths was compared between patients with T1–2N0M0 BC and the general population (Table 2). In general, patients with T1–2N0M0 of breast cancer had higher risks of all-cause death and CVD-specific death than the general population. The highest number of overall mortality and CVD-specific mortality occurred in patients in HR+/HER2− BC, whereas the SMRs of HR−/HER2+ and triple-negative BC were higher than other subtypes. Compared to the general population, younger patients with T1–2N0M0 BC had a higher risk of CVD mortality, and the SMR declined for patients diagnosed at an older age: for example, HR+/HER2− BC patients >85-year olds had an SMR of 2.66 (95% CI: 2.58–2.74, *P* < .0001) versus those <45 years had an SMR of 49.57 (95% CI: 44.11–47.88, *P* < .0001). Additionally, the SMRs of CVD mortality increased significantly over time: for example, triple-negative BC patients had an SMR of 2.49 (95% CI: 1.45–3.99, *P* < .0001) within the first year of diagnosis and the SMR increased to 11.50 (95% CI: 8.01–16.00, *P* < .0001) after 5-year follow-up.

**Table 2:** Standardized mortality ratio among patients with T1–2N0M0 breast cancer based on molecular subtypes.

	HR+/HER2+	HR−/HER2+	HR+/HER2−	HR−/HER2−
Overall mortality	7.18 (6.82–7.56)	8.79 (8.13–9.50)	5.23 (5.15–5.32)	9.90 (9.54–10.26)
Time after initial diagnosis				
≤1	4.37 (3.79–5.02)	5.14 (4.13–6.32)	2.50 (2.37–2.64)	5.24 (4.72–5.81)
1–3	5.58 (5.09–6.10)	7.95 (7.01–8.98)	3.88 (3.76–4.00)	9.32 (8.80–9.87)
3–5	8.86 (8.05–9.72)	10.73 (9.22–12.40)	6.39 (6.20–6.59)	12.82 (11.98–13.71)
>5	15.12 (13.59–16.78)	17.58 (14.54–21.07)	11.89 (11.52–12.27)	16.73 (15.27–18.28)
Age at diagnosis, y				
≤65	52.22 (47.21–57.62)	61.73 (53.82–70.46)	45.97 (44.11–47.88)	64.46 (60.75–68.34)
65–85	10.23 (9.49–11.02)	10.99 (9.76–12.34)	8.33 (8.14–8.53)	11.73 (11.09–12.39)
>85	2.97 (2.69–3.28)	3.20 (2.69–3.77)	2.66 (2.58–2.74)	3.10 (2.84–3.37)
Race				
White	6.61 (6.24–7.00)	8.25 (7.55–9.00)	4.93 (4.84–5.02)	8.80 (8.43–9.18)
Black	9.35 (8.12–10.70)	11.01 (8.94–13.42)	7.50 (7.11–7.90)	15.25 (14.08–16.49)
Other	19.23 (15.25–23.93)	12.55 (8.97–17.09)	8.58 (7.93–9.26)	14.42 (12.03–17.15)
CVD mortality	4.78 (3.88–5.84)	4.27 (3.05–5.82)	3.78 (3.58–4.00)	4.95 (4.20–5.80)
Time after initial diagnosis				
≤1	3.37 (1.89–5.56)	2.19 (0.60–5.62)	1.67 (1.37–2.01)	2.49 (1.45–3.99)
1–3	3.52 (2.38–5.03)	4.01 (2.25–6.62)	2.72 (2.46–3.01)	3.59 (2.66–4.75)
3–5	5.66 (3.76–8.18)	3.96 (1.90–7.28)	4.49 (4.07–4.95)	6.98 (5.23–9.13)
>5	10.15 (6.50–15.10)	8.60 (4.29–15.39)	8.80 (7.94–9.72)	11.50 (8.01–16.00)
Age at diagnosis, y				
≤65	32.12 (21.51–46.13)	45.47 (25.99–73.85)	37.52 (32.92–42.58)	36.35 (26.80–48.19)
65–85	9.25 (7.87–10.81)	8.99 (6.81–11.65)	7.39 (7.04–7.76)	9.19 (7.96–10.55)
>85	2.79 (2.33–3.31)	2.85 (2.03–3.90)	2.57 (2.45–2.70)	2.63 (2.25–3.06)
Race				
White	4.45 (3.91–5.04)	5.18 (4.17–6.35)	3.81 (3.68–3.95)	4.28 (3.83–4.77)
Black	7.20 (5.34–9.49)	7.20 (3.59–12.88)	6.31 (5.67–7.01)	8.34 (6.56–10.46)
Other	9.23 (5.56–14.42)	7.57 (3.27–14.91)	6.39 (5.45–7.44)	7.09 (4.20–11.20)

### 3.4 Risk of cause-specific mortality among patients

Univariate and multivariate analyses were performed to identify potential predictors of overall death, and deaths from breast cancer and CVD. Table 3 demonstrated the hazard ratios (HRs) stratified by subgroups. Older age at diagnosis was significantly associated with higher risk of all-cause of mortality, BC-specific mortality, and CVD-specific mortality. Notably, the result of multivariate analysis showed that the prognostic effect of age was significantly higher for CVD-specific death (HR = 209.943, 95% CI: 118.591–371.666) than that for BC-specific death (HR = 5.644, 95% CI: 4.893–6.511). In terms of cancer subtype, patients with triple-negative BC had highest risk of overall mortality (HR = 1.542, 95% CI: 1.454–1.635), BC-specific mortality (HR = 2.145, 95% CI: 1.962–2.345), and CVD-specific mortality (HR = 1.515, 95% CI: 1.213–1.892). Additionally, poor differentiation grade, advanced T stage, and advanced N stage were significantly associated with higher overall mortality and BC specific mortality. T2 stage and black race were associated with higher CVD-mortality rate.

**Table 3:** Univariate and multivariate analysis of prognostic factors for cause-specific mortality among patients with T1–2N0M0 breast cancer.

	Overall mortality		Breast cancer-specific mortality		CVD mortality	
Variable	HR^1^ (95% CI)	Adj HR^1^ (95% CI)	HR^1^ (95% CI)	Adj HR^1^ (95% CI)	HR^1^ (95% CI)	Adj HR^1^ (95% CI)
Age, y						
≤45	Ref	Ref	Ref	Ref	Ref	Ref
45–65	1.019 (0.921–1.126)	1.206 (1.090–1.334)	0.637 (0.566–0.717)	0.910 (0.808–1.025)	4.097 (2.296–7.312)	4.426 (2.479–7.901)
65–85	3.163 (2.875–3.480)	4.057 (3.683–4.469)	0.953 (0.848–1.071)	1.685 (1.496–1.897)	29.660 (16.803–52.357)	33.176 (18.779–58.612)
>85	15.175 (13.719–16.785)	18.057 (16.306–1.995)	3.587 (3.117–4.128)	5.644 (4.893–6.511)	198.926 (112.470–351.841)	209.943 (118.591–371.666)
Race						
White	Ref	Ref	Ref	Ref	Ref	Ref
Black	1.270 (1.198–1.347)	1.320 (1.245–1.401)	1.867 (1.703–2.046)	1.442 (1.314–1.584)	1.027 (0.900–1.173)	1.273 (1.114–1.456)
Other	0.553 (0.507–0.603)	0.652 (0.598–0.710)	0.647 (0.557–0.750)	0.654 (0.563–0.759)	0.531 (0.443–0.637)	0.709 (0.591–0.851)
Grade						
Well	Ref	Ref	Ref	Ref	Ref	Ref
Moderately	1.211 (1.152–1.272)	1.114 (1.060–1.172)	2.390 (2.100–2.720)	1.988 (1.745–2.265)	1.056 (0.961–1.161)	0.989 (0.898–1.089)
Poorly	1.691 (1.607–1.780)	1.472 (1.386–1.563)	7.099 (6.277–8.029)	3.998 (3.491–4.579)	0.906 (0.812–1.012)	0.923 (0.812–1.050)
Undifferentiated	2.088 (1.545–2.823)	2.073 (1.531–2.807)	10.077 (6.699–15.160)	6.469 (4.283–9.771)	0.502 (0.162–1.560)	0.586 (0.188–1.825)
Molecular type						
HR+/HER2−	Ref	Ref	Ref	Ref	Ref	Ref
HR+/HER2+	0.959 (0.893–1.030)	1.016 (0.944–1.093)	1.416 (1.250–1.604)	0.999 (0.879–1.135)	0.873 (0.751–1.014)	1.215 (1.041–1.418)
HR−/HER2+	1.177 (1.062–1.304)	1.137 (1.022–1.264)	2.533 (2.183–2.939)	1.454 (1.246–1.697)	1.226 (1.086–1.358)	1.391 (1.209–1.601)
HR−/HER2−	1.785 (1.696–1.878)	1.542 (1.454–1.635)	4.354 (4.032–4.701)	2.145 (1.962–2.345)	1.439 (1.315–1.557)	1.515 (1.213–1.892)
T stage						
T1	Ref	Ref	Ref	Ref	Ref	Ref
T2	2.127 (2.046–2.211)	1.860 (1.786–1.937)	4.018 (3.749–4.305)	2.732 (2.542–2.936)	1.697 (1.561–1.846)	1.610 (1.475–1.757)

## 4 Discussion

In this study, using the SEER database, we comprehensively analyzed the mortality patterns of T1–2N0M0 BC patients with different molecular subtypes in the United States. Although BC remains the leading cause of mortality initially after diagnosis, with approximately 50% mortalities due to breast cancer occurring within the first year of diagnosis, its frequency gradually declined as time passed after diagnosis to be surpassed by noncancer causes of death. CVDs were among the leading causes of noncancer mortality during the follow-up period after diagnosis, and became more frequent overtime. Additionally, in the present study, several clinicopathological factors were identified as independent predictors of overall death, BC-specific death, and CVD-specific death.

The survival prognosis of BC patients had significantly improved throughout the past 20 years,^[10]^ which could be attributed to the remarkable progress in the treatment of T1–2N0M0 BC, including surgical resection, radiation therapy, and multiple combinations of drugs.^[11–13]^ Consequently, BC patients are living longer to an extent at which noncancer mortalities significantly influence their overall survival. Therefore, it is critical to put insight on the mortalities from noncancer diseases when counseling the survivorship and prognosis for BC patients.

In the present study, SMRs calculation provides essential population-based data to assist clinicians in identifying BC patients at higher risks of cause-specific mortality compared to the general population. Notably, deaths from CVDs, including heart diseases, cardiomyopathy, cerebrovascular disease, and other CVDs, constituted the predominant causes of noncancer mortality. The combination of anthracyclines and new-generation targeted drugs (eg, trastuzumab) remains a key strategy in the treatment of breast cancer. Previous studies have reported that cardiotoxicity is a severe side-effect of these agents, involving irreversible cardiomyocyte mortality owing to the production of reactive oxygen species within the cardiomyocytes.^[14,15]^ Additionally, previous studies reported that endocrine treatment for BC was associated with the increased risk of pulmonary embolism, venous thromboembolism, and stroke.^[16]^ this effect of adjuvant antiestrogen treatment for breast cancer may also partially explain the CVD mortality that we observed.

In the case of radiotherapy, an elevated risk of ischemic heart disease may occur after cardiac exposure. A reasonable explanation is that radiation treatment induces injury to the micro-vessels in the myocardium or aggravates the progression of atherosclerosis in the macro-vessel.^[17]^ Notably, the risk of ischemic heart disease elevated linearly with increasing mean whole heart radiation dose.^[18]^ A population-based study of CVD in women receiving radiotherapy for breast cancer revealed that the rates of major coronary events elevated by 7.4%/ per Gy mean whole heart radiation dose, and this increase initiated within a few years after radiation exposure and continued for at least 2 decades.^[19]^

With respect to the prognostic factors of patients with T1–2N0M0 breast cancer, we observed that age at diagnosis was the strongest predictor for mortality in T1–2N0M0 BC patients, especially for CVD mortality, of which the HRs increased sharply with age. Poor differentiation grade was significantly associated with increased risk of death from overall cause and breast cancer, whereas these associations were not statistically significant in mortality from CVD. Additionally, patients with triple-negative BC (HR−/HER2−) had the highest risk of mortality from all cause, breast cancer, and CVDs, followed by HR−/HER2+ BC.

In the present study, we included a population-based patient cohort and long-term follow-up of survival with ascertainment of causes of mortality provided by the SEER database. However, apart from the retrospective nature of our study, there are still several limitations that need to be emphasized. First, the SEER program lacks the information regarding height and weight, medical comorbidities, and performance status, so we are unable to specify the potential prognostic effect of these factors on cause-specific death. For example, previous studies have demonstrated that obesity and overweight adversely impacted CVD mortality trends and were significantly associated with the incidence of breast cancer.^[20,21]^ The raised burden of overweight and obesity presented increasing challenge to T1–2N0M0 BC survivors who were increasingly susceptible to CVD mortality with age. These factors may participate in modeling the mortality landscape of T1–2N0M0 breast cancer, highlighting the need for further investigation. Second, due to the lack of detailed data on therapy, our study failed to reveal the impact of therapies on the trend of cause-specific mortality. What's more, the CODs in SEER was extracted from death certificates, which might suffer accuracy issues, nevertheless, we included all eligible patients and “big data” might help to mitigate this limitation.

## 5 Conclusion

In this large population-based study, the mortality pattern of T1–2N0M0 breast cancer survivors was investigated. Compared with the general population, breast cancer patients were at a higher risk of non-cancer mortality, especially cardiovascular death, and this risk increased with time. In addition, older age at diagnosis, black race, and aggressive histologic subtypes were identified to be independent predictors for CVD-specific mortality. Our findings recommended T1–2N0M0 BC survivors with these adverse factors for early detection and more efficient CVD care.

## Author contributions

**Conceptualization:** Daoliang Wang.

**Data curation:** Daoliang Wang, Lijun Zhang.

**Formal analysis:** Daoliang Wang, Lijun Zhang.

**Funding acquisition:** Liang Yi.

**Investigation:** Liang Yi.

**Methodology:** Daoliang Wang, Liang Yi, Lijun Zhang.

**Project administration:** Daoliang Wang, Liang Yi, Lijun Zhang.

**Resources:** Liang Yi, Zhuo Wang.

**Software:** Liang Yi, Lijun Zhang, Zhuo Wang.

**Supervision:** Daoliang Wang, Zhuo Wang.

**Validation:** Liang Yi, Lijun Zhang, Zhuo Wang.

**Visualization:** Daoliang Wang, Liang Yi, Lijun Zhang, Zhuo Wang.

**Writing – original draft:** Daoliang Wang, Liang Yi, Zhuo Wang.

**Writing – review & editing:** Daoliang Wang, Lijun Zhang, Zhuo Wang.
